# Supplementary material for: Megaripple mechanics: bimodal transport ingrained in bimodal sands
Source: Nat Commun. 2022 Jan 10;13:162. doi: 10.1038/s41467-021-26985-3 (PMC8748662; doi:10.1038/s41467-021-26985-3)
Supplement: Supplementary file 1 — Supplementary Information [file 41467_2021_26985_MOESM1_ESM.pdf]

Megaripple mechanics: bimodal transport ingrained in bimodal sands — Supplementary Information

Katharina Tholen[1], Thomas Pähtz[2*], Hezi Yizhaq[3] , Itzhak Katra[4*], Klaus Kroy[1*a)]

[1]*Institute for Theoretical Physics, Leipzig University, Leipzig, Germany.* [2]*Institute of Port, Coastal and Offshore Engineering, Ocean College, Zhejiang University, 866 Yu Hang Tang Road, 310058 Hangzhou, China.* [3]*Department of Solar Energy and Environmental Physics, Blaustein Institutes for Desert Research, Ben-Gurion University of the Negev, Sede Boqer Campus, Be'er Sheva, Israel.* [4]*Department of Geography and Environmental Development, Ben-Gurion University of the Negev, Be'er Sheva, Israel.*

(Dated: 30 October 2021)

g	gravitational constant
$\tilde{g} \equiv (1-1/s)g$	buoyancy-reduced gravitational constant
ρ_p (ρ_a)	grain (fluid) density
$s \equiv \rho_\mathrm{p}/\rho_\mathrm{a}$	grain–fluid density ratio
ν_a	kinematic fluid viscosity
τ	shear stress of a flow applied onto the sand surface
$d^{(\mathrm{c})}$, $d^{(\mathrm{f})}$	coarse and fine grain diameters (of bidisperse sand)
$\Theta \equiv \tau/(\rho_\mathrm{p}\tilde{g}d^{(\mathrm{f,c})})$	fine-(coarse-)grain Shields number
$\mathrm{Ga}^{(\mathrm{f})} \equiv \sqrt{s\tilde{g}(d^{(\mathrm{f})})^3}/\nu_\mathrm{a}$	fine-grain Galileo number
$\tau_\mathrm{t}(d^{(\mathrm{f})})$, $\tau_\mathrm{t}(d^{(\mathrm{c})})$	saltation threshold of the fine and coarse grains
$\tau_\mathrm{r}(d^{(\mathrm{c})})$	reptation threshold of coarse grains
$\max(d^{(\mathrm{f})})$, $\max(d^{(\mathrm{c})})$	size of biggest saltating fine grains and reptating coarse grains in a continuous GSD
$\tau_\mathrm{t}(\max(d^{(\mathrm{f,c})}))$	saltation threshold of biggest saltating fine (coarse) grains
$\tau_\mathrm{r}(\max(d^{(\mathrm{c})}))$	reptation threshold of biggest reptating coarse grains
V_s	dimensionless settling velocity
μ_r	rebound momentum restitution coefficient
$C_z \equiv v_{\uparrow z}^{(\mathrm{f})}/\overline{v_x}^{(\mathrm{f})}$	proportionality constant between the vertical rebound velocity $v_{\uparrow z}^{(\mathrm{f})}$ and the mean horizontal velocity $\overline{v_x}^{(\mathrm{f})}$ of the fine grain
z_0	bed roughness height

Table S1 Mathematical symbols used in the main text. For further mathematical symbols used in the Method Section, see Notation Section in Ref.[1].

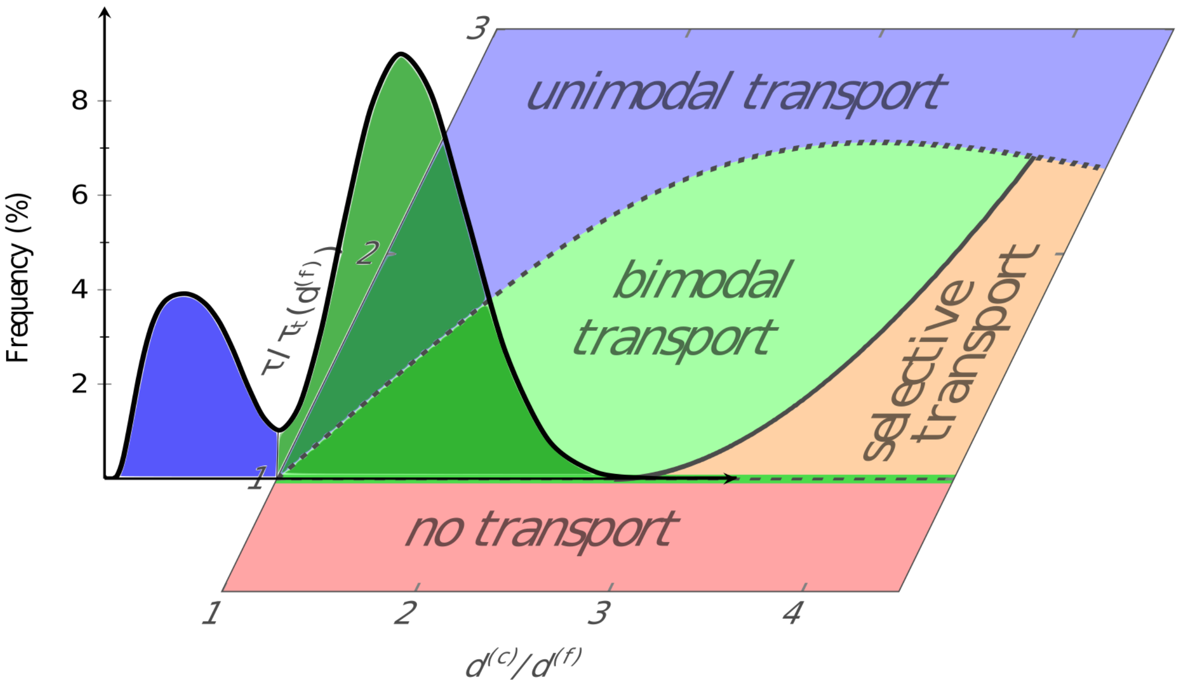

Fig. S1 **Connecting megaripple morphodynamics to crest GSDs:** Based on the notion that megaripple formation is due to bimodal transport, where fine-grain saltation drives coarse-grain reptation, a phase diagram of all relevant possible transport modes can be calculated for idealized bidisperse sand (horizontal diagram). It is parametrized by the coarse-fine grain size ratio $d^{(c)}/d^{(f)}$ and the wind strength τ normalized by the fine-grain saltation threshold $\tau_t(d^{(f)})$. It can be read as a catalogue of dynamical modes of megaripple evolution. Grafting the bimodal GSD vertically onto the phase diagram, as discussed around Fig. 3 of the main text, reveals the mechanistic origin of our main quantitative prediction: a well conserved log-scale width of the coarse-grain peak (green shaded) of all GSDs evolving under similar ambient conditions.

S1. CRITICAL ENERGY

To quantify the reptation threshold (Method Sec. M1.A) we estimate the minimum kinetic energy $E_{\rm crit}$ a coarse bed grain needs to leave its trap and get permanently displaced. Consider a coarse grain resting on a two-dimensional granular packing. As already detailed in Sec. M1.A, we assume the grain has received the momentum $m^{(c)}\mathbf{v}^{(c)} = m^{(c)}(v_x^{(c)}, v_z^{(c)})$ by an impact of the saltating fine grain. Assuming the jump to be undisturbed by the surrounding air, a minimum displacement $(\Delta x, \Delta z) = (d^{(c)}/2, z_b)$ is required for the grain to escape its

trap, with $z_b = (1 - \sin\Psi)d^{(c)}$ and the pocket angle Ψ characteristic of the corresponding bed arrangement (see Fig. 3 in Ref.[1]). In the vertical motion, the grain will first arrive at its topmost position $z_m = v_z^{(c)^2}/(2\tilde{g}) \geq z_b$ after the time $t_m = v_z^{(c)}/\tilde{g}$ and then fall to the position z_b in the additional time $t_f = \sqrt{2(z_m - z_b)/\tilde{g}}$. In the total flight time $t_m + t_f$, it must therefore travel the critical horizontal distance $d^{(c)}/2$. Hence,

$$\left(\sqrt{\frac{\left(v_z^{(c)}\right)^2}{\tilde{g}^2} - \frac{2z_b}{\tilde{g}}} + \frac{v_z^{(c)}}{\tilde{g}}\right) v_x^{(c)} = \frac{d^{(c)}}{2}. \tag{S1}$$

Minimizing the grain's kinetic energy under the constraint S1 yields

$$\frac{E_{\text{crit}}}{m^{(c)}\tilde{g}d^{(c)}} = \frac{z_b}{2d^{(c)}}\left(1 + \sqrt{1 + \left(\frac{2z_b}{d^{(c)}}\right)^{-2}}\right). \tag{S2}$$

Consequently, the (non-dimensionalized) critical energy depends only on the given pocket angle Ψ. For a two-dimensional hexagonal arrangement, $\Psi = 60°$.

S2. GENERALIZED REBOUND MODEL

As the periodic saltation model (Sec. M1.C) was originally constructed for monodisperse sand, it has to be revised for the scenario in which fine grains saltate along a bed of coarse grains. A considerable part of this modification is to provide a generalized relation quantifying grain-bed collisions between an impacting grain and a bed grain of different diameter ($d^{(c)} \neq d^{(f)}$). For this purpose, we employ the two-dimensional rebound model introduced by Lämmel and Kroy[2], and improve it in such a manner that it can be analytically solved.

We first summarize the formal description of the rebound process. In the following, all lengths given in units of the mean diameter $d \equiv (d^{(c)} + d^{(f)})/2$ are labelled by a hat. For example,

$$\hat{d}^{(c,f)} = \frac{2}{1 + \frac{d^{(f,c)}}{d^{(c,f)}}}. \tag{S3}$$

Consider a coarse grain resting in a pocket of a two-dimensional coarse-grain packing and a fine grain with the impact velocity $\mathbf{v}_\downarrow^{(f)}$ and rebound velocity $\mathbf{v}_\uparrow^{(f)}$ rebounding from the coarse grain (see Fig. S2). The rebound velocities are modeled via momentum conservation

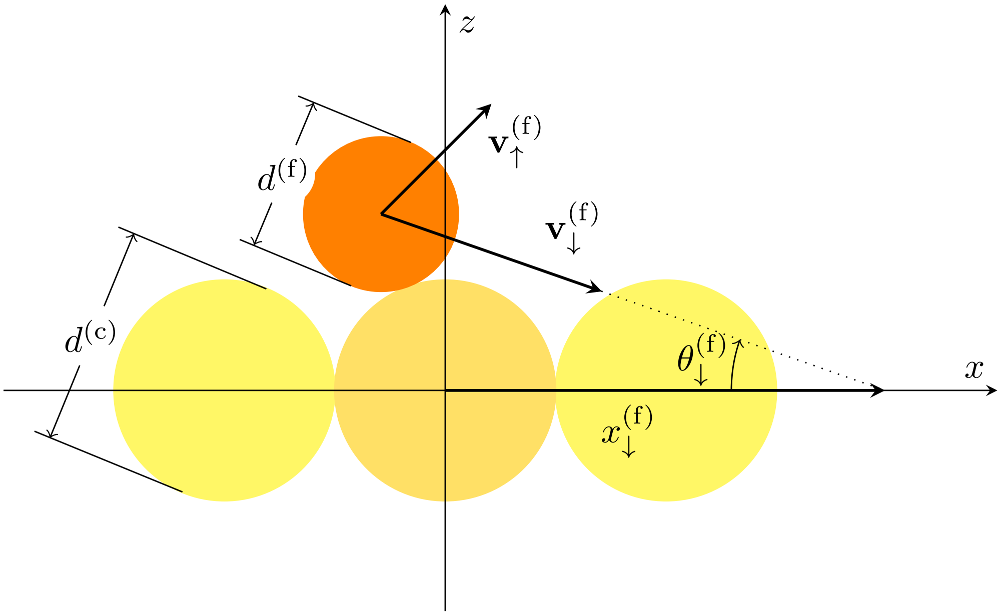

FIG. S2 **Schematic of two-dimensional collision model:** the spherical impactor of diameter $d^{(\mathrm{f})}$ strikes the homogeneous flat bed with impact velocity $\mathbf{v}_{\downarrow}^{(\mathrm{f})}$ and bounces off the target bed grain of diameter $d^{(\mathrm{c})}$ located at the origin. Without the bed grains, the impactor would cross the x-axis at $x_{\downarrow}^{(\mathrm{f})}$. Figure adapted from Fig 1. in Ref.[2].

and energy dissipation as appropriate for an inelastic collision between the impactor and the bed grain (see Eq. 9a and 9b in Ref.[2])

$$\frac{v_{\uparrow x}^{(\mathrm{f})}}{|\mathbf{v}_{\downarrow}^{(\mathrm{f})}|} = (\alpha+\beta)\hat{x}_{\downarrow}^{(\mathrm{f})}\sin^2\theta_{\downarrow}^{(\mathrm{f})}\sqrt{1-\left(\hat{x}_{\downarrow}^{(\mathrm{f})}\right)^2\sin^2\theta_{\downarrow}^{(\mathrm{f})}} + \left[(\alpha+\beta)\left(\hat{x}_{\downarrow}^{(\mathrm{f})}\right)^2\sin^2\theta_{\downarrow}^{(\mathrm{f})} - \alpha\right]\cos\theta_{\downarrow}^{(\mathrm{f})}\,, \tag{S4a}$$

$$\frac{v_{\uparrow z}^{(\mathrm{f})}}{|\mathbf{v}_{\downarrow}^{(\mathrm{f})}|} = \left[\alpha - (\alpha+\beta)\left(\left(\hat{x}_{\downarrow}^{(\mathrm{f})}\right)^2\sin^2\theta_{\downarrow}^{(\mathrm{f})} - \hat{x}_{\downarrow}^{(\mathrm{f})}\cos\theta_{\downarrow}^{(\mathrm{f})}\sqrt{1-\left(\hat{x}_{\downarrow}^{(\mathrm{f})}\right)^2\sin^2\theta_{\downarrow}^{(\mathrm{f})}}\right)\right]\sin\theta_{\downarrow}^{(\mathrm{f})}, \tag{S4b}$$

in terms of the effective restitution coefficients for the normal (α) and the tangential(β) velocity component of the rebounding grain, respectively:

$$\alpha = \frac{1+\epsilon}{1+\mu} - 1 \quad \text{and} \quad \beta = 1 - \frac{(2/7)(1-\nu)}{1+\mu}\,, \quad \text{with } \mu = \left[\left(\frac{\hat{d}^{(\mathrm{c})}}{\hat{d}^{(\mathrm{f})}}\right)^3 + 1/\epsilon\right]^{-1}. \tag{S5}$$

In the collision process, energy dissipation is determined by the microscopic restitution coefficients ϵ and ν for the normal and tangential component of the relative impact velocity

of the collision partners. Furthermore, the semi-empirical parameter μ accounts for the effect of non-binary collisions when the impactor is of comparable (or larger) size than the bed grain. The mean value of functions $A[\mathbf{v}_\uparrow^{(\mathrm{f})}(\mathbf{v}_\downarrow^{(\mathrm{f})}, \hat{x}_\downarrow^{(\mathrm{f})})]$ of the rebound velocity is obtained by averaging over all possible impact positions $x_\downarrow^{(\mathrm{f})}$:

$$\overline{A}(\mathbf{v}_\downarrow^{(\mathrm{f})}) = \frac{1}{\hat{x}_{max} - \hat{x}_{min}} \int_{\hat{x}_{min}}^{\hat{x}_{max}} \mathrm{d}\hat{x}_\downarrow^{(\mathrm{f})} \, A[\mathbf{v}_\uparrow^{(\mathrm{f})}(\mathbf{v}_\downarrow^{(\mathrm{f})}, \hat{x}_\downarrow^{(\mathrm{f})})], \tag{S6}$$

where $\hat{x}_{min}$ is determined from the contact condition between the impactor and the bed grain

$$\hat{x}_{min} = \begin{cases} \csc\theta_\downarrow^{(\mathrm{f})} - \hat{d}^{(\mathrm{c})}\,, & \text{if } 2\sin\theta_1 < \hat{d}^{(\mathrm{c})}\,, \\ \cot\theta_\downarrow^{(\mathrm{f})}\sqrt{1-(\hat{d}^{(\mathrm{c})}/2)^2} - \hat{d}^{(\mathrm{c})}/2\,, & \text{else.} \end{cases} \tag{S7}$$

The maximum impact position $\hat{x}_{max}$ is obtained by neglecting secondary collisions with $v_{\uparrow z}^{(\mathrm{f})} < 0$:

$$\frac{1}{1+\beta/\alpha} < \hat{x}_{max}^2 \sin^2\theta_\downarrow^{(\mathrm{f})} - \hat{x}_{max}\cos\theta_\downarrow^{(\mathrm{f})}\sqrt{1-\hat{x}_{max}^2\sin^2\theta_\downarrow^{(\mathrm{f})}}. \tag{S8}$$

While the original model[2] provided the mean total and vertical restitution coefficient $\overline{e}$ and $\overline{e_z}$ by explicitly computing the average of their statistical distributions (i.e., $A = e$ and $A = e_z$), we here determine physically more appropriate mean values deduced from the mean vertical and horizontal rebound velocity. More precisely:

$$\begin{aligned} \overline{e_{v_x}} \equiv \frac{\overline{v_{\uparrow x}^{(\mathrm{f})}}}{|\mathbf{v}_\downarrow^{(\mathrm{f})}|} =& \frac{1}{\hat{x}_{max} - \hat{x}_{min}} \frac{1}{3} \Bigg[-(\alpha+\beta)\left(1 - \left(\hat{x}_\downarrow^{(\mathrm{f})}\right)^2 \sin^2\theta_\downarrow^{(\mathrm{f})}\right)^{3/2} \\ & + \hat{x}_\downarrow^{(\mathrm{f})}\cos\theta_\downarrow^{(\mathrm{f})}\left(-3\alpha + (\alpha+\beta)\left(\hat{x}_\downarrow^{(\mathrm{f})}\right)^2\sin^2\theta_\downarrow^{(\mathrm{f})}\right)\Bigg]_{\hat{x}_{min}}^{\hat{x}_{max}}, \end{aligned} \tag{S9a}$$

$$\begin{aligned} \overline{e_{v_z}} \equiv \frac{\overline{v_{\uparrow z}^{(\mathrm{f})}}}{|\mathbf{v}_\downarrow^{(\mathrm{f})}|} =& \frac{\sin\theta_\downarrow^{(\mathrm{f})}}{\hat{x}_{max} - \hat{x}_{min}} \Bigg[\alpha\hat{x}_\downarrow^{(\mathrm{f})} - \frac{1}{3}(\alpha+\beta)\Bigg(\left(\hat{x}_\downarrow^{(\mathrm{f})}\right)^3 \sin^2\theta_\downarrow^{(\mathrm{f})} \\ & + \cot\theta_\downarrow^{(\mathrm{f})}\csc\theta_\downarrow^{(\mathrm{f})}(1 - \left(\hat{x}_\downarrow^{(\mathrm{f})}\right)^2\sin^2\theta_\downarrow^{(\mathrm{f})})^{3/2}\Bigg)\Bigg]_{\hat{x}_{min}}^{\hat{x}_{max}}, \end{aligned} \tag{S9b}$$

and

$$\overline{e} = \sqrt{\overline{e_{v_x}}^2 + \overline{e_{v_z}}^2}\,, \tag{S10a}$$

$$\overline{e_z} = \overline{e_{v_z}} \sin^{-1}\theta_\downarrow^{(\mathrm{f})}\,. \tag{S10b}$$

In contrast to the original model, this method provides an analytical solution for all impact geometries. Setting the values of the (effective) microscopic restitution coefficients $\epsilon = 0.78$

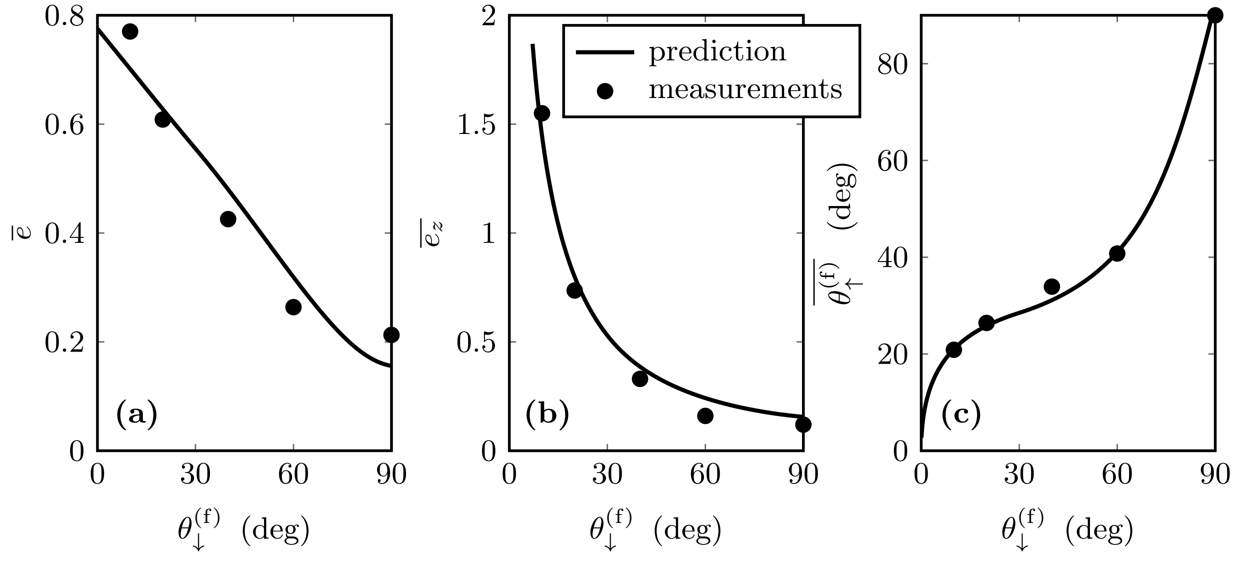

Fig. S3 **Comparison of the rebound model with literature data:** The measured[3] averaged key characteristics of the rebound process (symbols) are the mean restitution coefficient $\overline{e}$ (a), Eq. S10a, mean vertical restitution coefficient $\overline{e_z}$ (b), Eq. S10b, and rebound angle $\overline{\theta_\uparrow^{(f)}} = \arcsin(\overline{e_z} \sin\theta_\downarrow^{(f)}/\overline{e})$ (c). They are well reproduced by the analytical two-dimensional model (solid line) over a wide range of impact angles $\theta_\downarrow^{(f)}$ (microscopic restitution coefficients $\epsilon = 0.78$ and $\nu = -0.13$).

and $\nu = -0.13$, the analytically evaluated two-dimensional model compares very well to the experimentally measured rebound averages for monodisperse granulates[3], as shown in Figure S3.

**Location**<br>$\nu_a\,[\mathrm{m^2\,s^{-1}}]$; $\rho_a\,[\mathrm{kg\,m^{-3}}]$; $\rho_p\,[\mathrm{kg\,m^{-3}}]$	$\max(d^{(f)})$<br>[µm]	$\max(d^{(c)})$<br>[µm]	References
**Nahal Kasuy, Israel**	530	1717	Yizhaq et al., 2019[4] (Fig. 3, "A09-MIN")
$1.63 \cdot 10^{-5}$; 1.135; 2689	564	1732	Yizhaq et al., 2019[4](Fig. 3, "A15-MIN")
	429	1254	Katra & Yizhaq, 2017[5] (Fig. 1, green line)
	577	1730	Katra & Yizhaq, 2017[5] (Fig. 9, red line)
	399	1126	Katra & Yizhaq, 2017[5] (Fig. 9, blue line)
	548	1739	Katra & Yizhaq, 2017[5] (Fig. 9, green line)
	430	1259	Yizhaq et al., 2012[6] (Fig. 6 "2.10.2008")
	264	689	Yizhaq et al., 2012[6] (Fig. 6 "23.10.2008")
	311	906	Yizhaq et al., 2012[6] (Fig. 6 "20.02.2009")
	305	1202	Yizhaq et al., 2012[6] (Fig. 6 "08.03.2009")
	335	1204	Yizhaq et al., 2012[6] (Fig. 6 "23.03.2009")
	237	682	Yizhaq et al., 2012[6] (Fig. 6 "31.03.2009")
	281	807	Yizhaq et al., 2012[6] (Fig. 6 "22.04.2009")
	297	778	Yizhaq et al., 2012[6] (Fig. 6 "12.05.2009")
	298	876	Yizhaq et al., 2012[6] (Fig. 6 "02.07.2009")
	237	682	Yizhaq et al., 2012[6] (Fig. 6 "13.03.2010")
	289	821	Yizhaq et al., 2012[6] (Fig. 6 "18.04.2010")
	322	858	Yizhaq et al., 2012[6] (Fig. 6 "28.08.2010")
	433	1591	Yizhaq et al., 2012[6] (Fig. 6 "14.12.2010")
	439	1649	Yizhaq et al., 2012[6] (Fig. 6 "09.02.2011")
	278	706	Yizhaq et al., 2012[6] (Fig. 6 "29.03.2011")
	563	1692	original data (Fig. S4, "1")
	548	1689	original data (Fig. S4, "2")
	528	1667	original data (Fig. S4, "3")
	553	1695	original data (Fig. S4, "4")
	588	1699	original data (Fig. S4, "5")
	555	1707	original data (Fig. S4, "6")

	560	1710	original data (Fig. S4, "7")
	562	1715	original data (Fig. S4, "8")
	570	1716	original data (Fig. S4, "9")
	553	1709	original data (Fig. S4, "10")
**Ktora, Israel**	488	1781	Katra & Yizhaq, 2017[5] (Fig. 1, red line)
$1.62 \cdot 10^{-5}$; 1.139; 2650	613	1710	original data (Fig. S5, "1")
	610	1714	original data (Fig. S5, "2")
	597	1716	original data (Fig. S5, "3")
	605	1718	original data (Fig. S5, "4")
**Yahel, Israel**	611	1706	original data (Fig. S6, "1")
$1.60 \cdot 10^{-5}$; 1.161; 2650	606	1715	original data (Fig. S6, "2")
	602	1713	original data (Fig. S6, "3")
	603	1714	original data (Fig. S6, "4")
	621	1716	original data (Fig. S6, "5")
	611	1714	original data (Fig. S6, "6")
	614	1716	original data (Fig. S6, "7")
**Wadi Rum, Jordan**	576	1737	Katra & Yizhaq, 2017[5] (Fig. 1, yellow line)
$1.68 \cdot 10^{-5}$; 1.095; 2650	583	1704	Katra & Yizhaq, 2017[5] (Fig. 10, "A0")
	594	1713	Katra & Yizhaq, 2017[5] (Fig. 10, "A5")
	599	1712	Katra & Yizhaq, 2017[5] (Fig. 10, "A10")
	601	1713	Katra & Yizhaq, 2017[5] (Fig. 10, "A15")
	604	1710	Katra & Yizhaq, 2017[5] (Fig. 10, "A20")
**Shanshan Desert, China**	446	1288	Katra & Yizhaq, 2017[5] (Fig. 1, blue line)
$1.57 \cdot 10^{-5}$; 1.182; 2650			
**Kumtagh Desert, China**	913	2963	Qian et al., 2012[7] (Fig. 4 P1, red points)
$1.77 \cdot 10^{-5}$; 1.022; 2650	982	3357	Qian et al., 2012[7] (Fig. 4 P2, red points)
	920	2949	Qian et al., 2012[7] (Fig. 4 P3, red points)
	1108	3655	Qian et al., 2012[7] (Fig. 4 P4, red points)
**Sossusvlei, Namibia**	653	1559	original data (Fig. S7, "1")
$1.61 \cdot 10^{-5}$; 1.150; 2650	687	1588	original data (Fig. S7, "2")

**Ladakh, India**	498	1690	original data (Fig. S8, "1")
$2.01 \cdot 10^{-5}$; 0.876; 2650	598	1724	original data (Fig. S8, "2")
**Wright Valley, Antarctica**	4830	16000	Gillies et al., 2012[8] (Fig. 14, "crest")
$1.70 \cdot 10^{-5}$; 1.074; 2080			
**White Sands, New Mexico**	1000	3200	Jerolmack et al., 2006[9] (Fig. 10, solid line)
$1.73 \cdot 10^{-5}$; 1.050; 2630			
**Wind tunnel**	289	821	Yizhaq et al., 2019[4] (Fig. 4, "$t = 9$")
$1.63 \cdot 10^{-5}$; 1.130; 2689	246	750	Yizhaq et al., 2019[4] (Fig. 4, "$t = 12$")
	331	1009	Yizhaq et al., 2019[4] (Fig. 4, "$t = 15$")
**Meridiani Planum, Mars**	650	3000	Jerolmack et al., 2006[9] (Fig. 7a)
$6.35 \cdot 10^{-4}$; 0.020; 4100			

TABLE S2: Extracted values of the left, $\max(d^{(f)})$, and right margin, $\max(d^{(c)})$, of the coarse-grain peak of measured GSDs collected from Refs.[4–9] and our original measurements of the crest GSDs (see Figs. S4-8 and Supplementary Data 1), and displayed in Fig. 5 of the main text.

**Location**	$\mathrm{mode}(d^{(f)})$ [µm]	$\mathrm{mode}(d^{(c)})$ [µm]	References
**Nahal Kasuy, Israel**	160	896	Yizhaq et al., 2019[4] (Fig. 3, "A09-MIN")
	195	1060	Yizhaq et al., 2019[4](Fig. 3, "A15-MIN")
	195	711	Katra & Yizhaq, 2017[5] (Fig. 1, green line)
	216	1060	Katra & Yizhaq, 2017[5] (Fig. 9, red line)
	160	645	Katra & Yizhaq, 2017[5] (Fig. 9, blue line)
	195	959	Katra & Yizhaq, 2017[5] (Fig. 9, green line)
	195	711	Yizhaq et al., 2012[6] (Fig. 6 "2.10.2008")
	160	291	Yizhaq et al., 2012[6] (Fig. 6 "23.10.2008")
	177	335	Yizhaq et al., 2012[6] (Fig. 6 "27.01.2009")
	160	478	Yizhaq et al., 2012[6] (Fig. 6 "20.02.2009")
	195	644	Yizhaq et al., 2012[6] (Fig. 6 "08.03.2009")
	177	583	Yizhaq et al., 2012[6] (Fig. 6 "23.03.2009")
	160	355	Yizhaq et al., 2012[6] (Fig. 6 "31.03.2009")

	160	433	Yizhaq et al., 2012[6] (Fig. 6 "22.04.2009")
	160	433	Yizhaq et al., 2012[6] (Fig. 6 "12.05.2009")
	177	433	Yizhaq et al., 2012[6] (Fig. 6 "02.07.2009")
	160	355	Yizhaq et al., 2012[6] (Fig. 6 "13.03.2010")
	145	433	Yizhaq et al., 2012[6] (Fig. 6 "18.04.2010")
	177	478	Yizhaq et al., 2012[6] (Fig. 6 "28.08.2010")
	177	785	Yizhaq et al., 2012[6] (Fig. 6 "14.12.2010")
	177	869	Yizhaq et al., 2012[6] (Fig. 6 "09.02.2011")
	177	355	Yizhaq et al., 2012[6] (Fig. 6 "29.03.2011")
	206	1010	original data (Fig. S4, "1")
	186	1010	original data (Fig. S4, "2")
	186	914	original data (Fig. S4, "3")
	186	1010	original data (Fig. S4, "4")
	186	1010	original data (Fig. S4, "5")
	186	1010	original data (Fig. S4, "6")
	169	1010	original data (Fig. S4, "7")
	186	1010	original data (Fig. S4, "8")
	186	1010	original data (Fig. S4, "9")
	186	1010	original data (Fig. S4, "10")
**Ktora, Israel**	291	958	Katra & Yizhaq, 2017[5] (Fig. 1, red line)
	374	1116	original data (Fig. S5, "1")
	374	1116	original data (Fig. S5, "2")
	322	1116	original data (Fig. S5, "3")
	322	1116	original data (Fig. S5, "4")
**Yahel, Israel**	456	1116	original data (Fig. S6, "1")
	291	1116	original data (Fig. S6, "2")
	355	1116	original data (Fig. S6, "3")
	322	1116	original data (Fig. S6, "4")
	216	1232	original data (Fig. S6, "5")
	239	1116	original data (Fig. S6, "6")

	216	1116	original data (Fig. S6, “7”)
**Wadi Rum, Jordan**	322	1059	Katra & Yizhaq, 2017[5] (Fig. 1, yellow line)
	433	959	Katra & Yizhaq, 2017[5] (Fig. 10, “A0”)
	355	1060	Katra & Yizhaq, 2017[5] (Fig. 10, “A5”)
	392	1060	Katra & Yizhaq, 2017[5] (Fig. 10, “A10”)
	392	1060	Katra & Yizhaq, 2017[5] (Fig. 10, “A15”)
	433	1000	Katra & Yizhaq, 2017[5] (Fig. 10, “A20”)
**Shanshan Desert, China**	216	711	Katra & Yizhaq, 2017[5] (Fig. 1, blue line)
**Kumtagh Desert, China**	113	2248	Qian et al., 2012[7] (Fig. 4 P1, red points)
	314	2248	Qian et al., 2012[7] (Fig. 4 P2, red points)
	143	2248	Qian et al., 2012[7] (Fig. 4 P3, red points)
	113	2850	Qian et al., 2012[7] (Fig. 4 P4, red points)
**Abra Pomez, Argentina**	1095	3054	Gough et al., 2020[10] (Fig. S6, “Megaripples”)
**Libyan desert, Libya**	146	1421	Bagnold, 1941[11] (Fig. 52)
	88	846	Bagnold, 1941[11] (Fig. 54)
**Sossusvlei, Namibia**	418	926	original data (Fig. S7, “1”)
	461	926	original data (Fig. S7, “2”)
**Ladakh, India**	456	914	original data (Fig. S8, “1”)
	145	1116	original data (Fig. S8, “2”)
**Wright Valley, Antarctica**	604	13656	Gillies et al., 2012[8] (Fig. 14, “crest”)
**Victoria Valley, Antarctica**	210	1189	Selby et al., 1974[12] (Fig. 11, “Whaleback dunes”)
**Askja Region, Iceland**	177	5657	Mountney & Russell, 2004[13] (Fig. 10b)
**Wind tunnel**	216	433	Yizhaq et al., 2019[4] (Fig. 4, “$t = 9$”)
	238	392	Yizhaq et al., 2019[4] (Fig. 4, “$t = 12$”)
	238	584	Yizhaq et al., 2019[4] (Fig. 4, “$t = 15$”)
	145	689	Hong et al., 2018 (Fig. 5, “$u^* = 0.51\,\mathrm{m\,s^{-1}}$”)
	134	723	Hong et al., 2018 (Fig. 5, “$u^* = 0.61\,\mathrm{m\,s^{-1}}$”)

	251	596	McKenna Neuman & Bèdard, 2017[15] (Fig. 1, "Texture 1")
	251	596	McKenna Neuman & Bèdard, 2017[15] (Fig. 1, "Texture 2")
	251	596	McKenna Neuman & Bèdard, 2017[15] (Fig. 1, "Texture 3")
	251	596	McKenna Neuman & Bèdard, 2017[15] (Fig. 1, "Texture 4")
	251	596	McKenna Neuman & Bèdard, 2017[15] (Fig. 1, "Texture 5")

Table S3: Characteristic fine and coarse grain size (modes), displayed in Fig. 1c of the main text. The modes are collected from Refs.[4–8,10–15] and extracted from our original measurements of the crest GSDs (see Figs. S4-8 and Supplementary Data 1). Note that GSDs for which the two peaks are indistinguishable were sorted out.

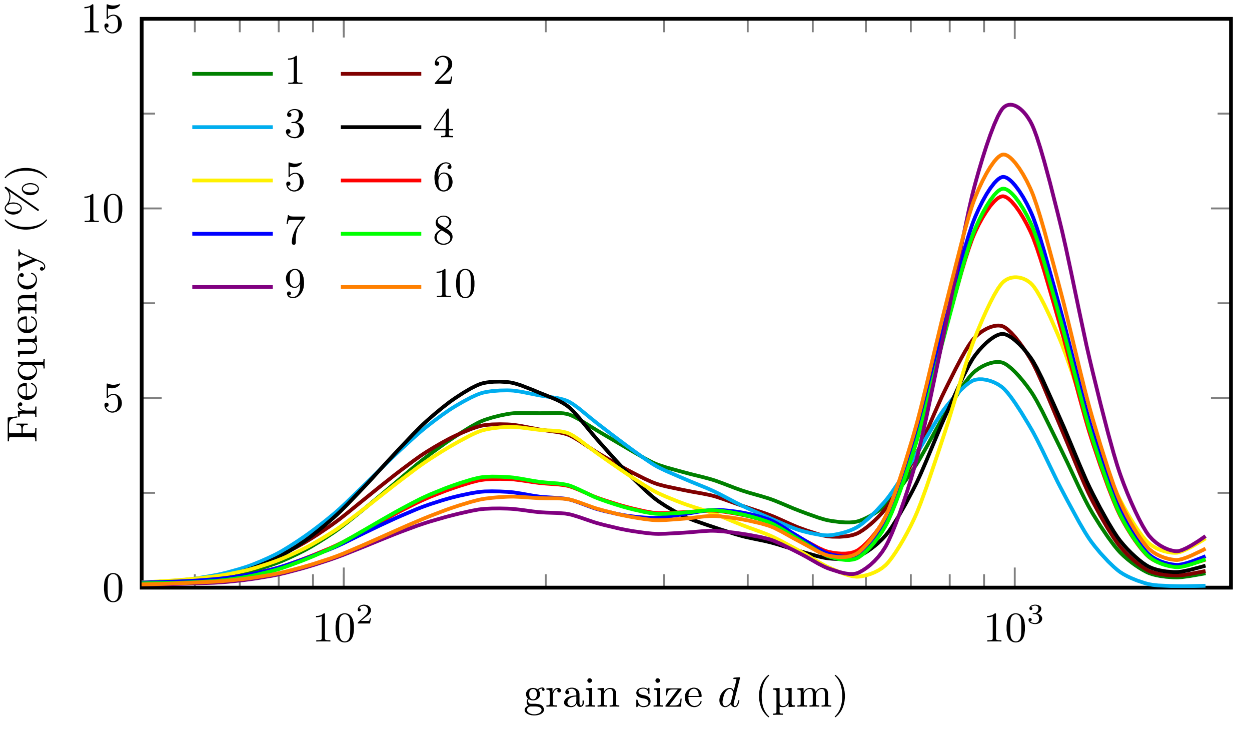

Fig. S4 Our measurements of the GSDs from Kasuy, Israel (see Fig. S9b).

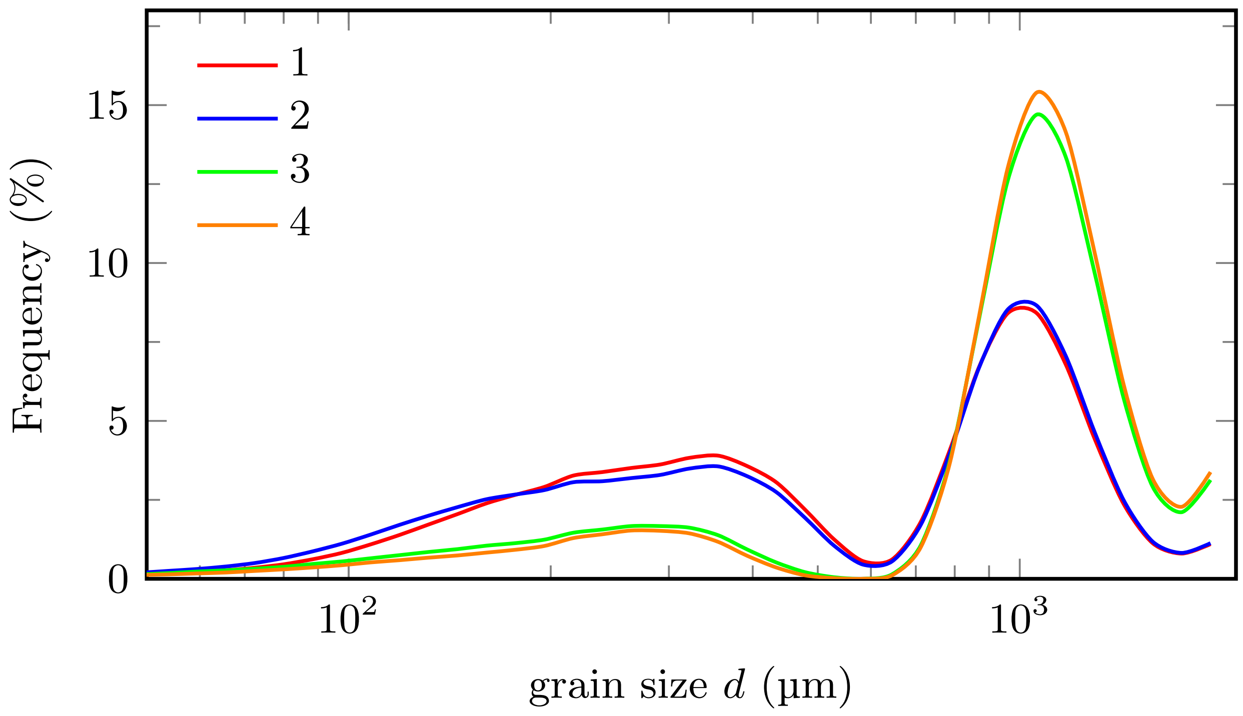

FIG. S5 Our measurements of the GSDs from Ktora, Israel (see Fig. S9a).

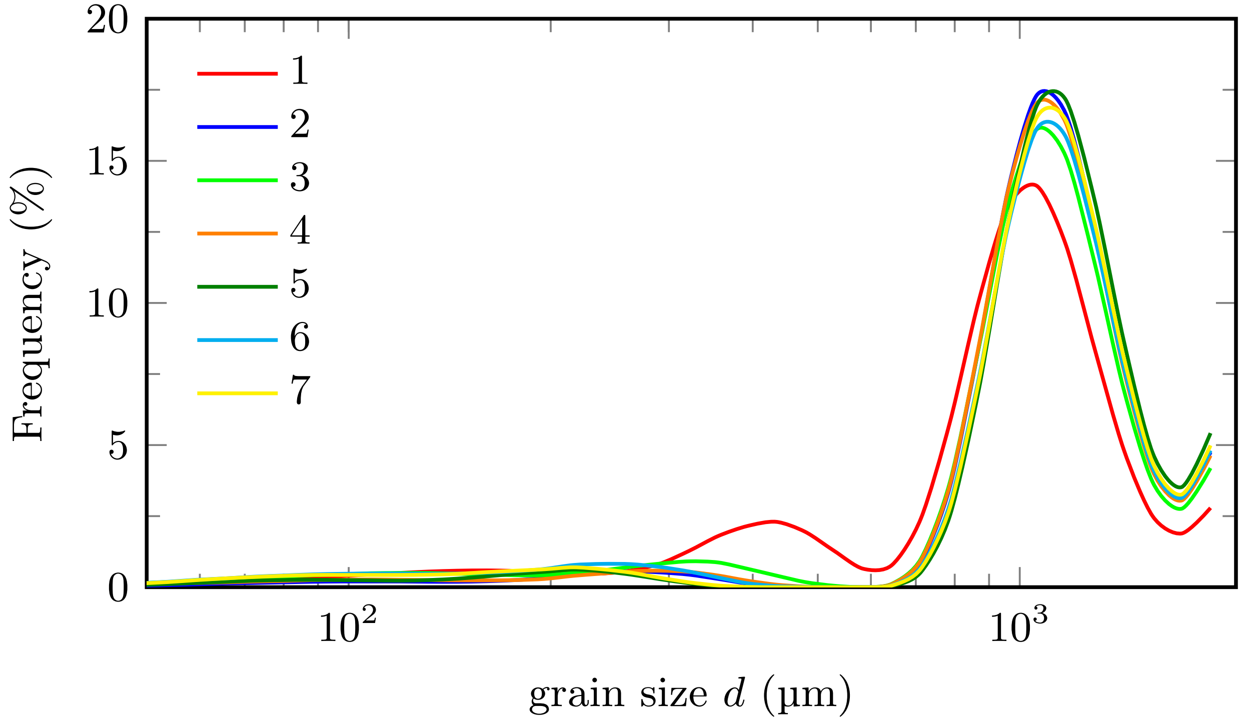

FIG. S6 Our measurements of the GSDs from Yahel, Israel (see Fig. S9c).

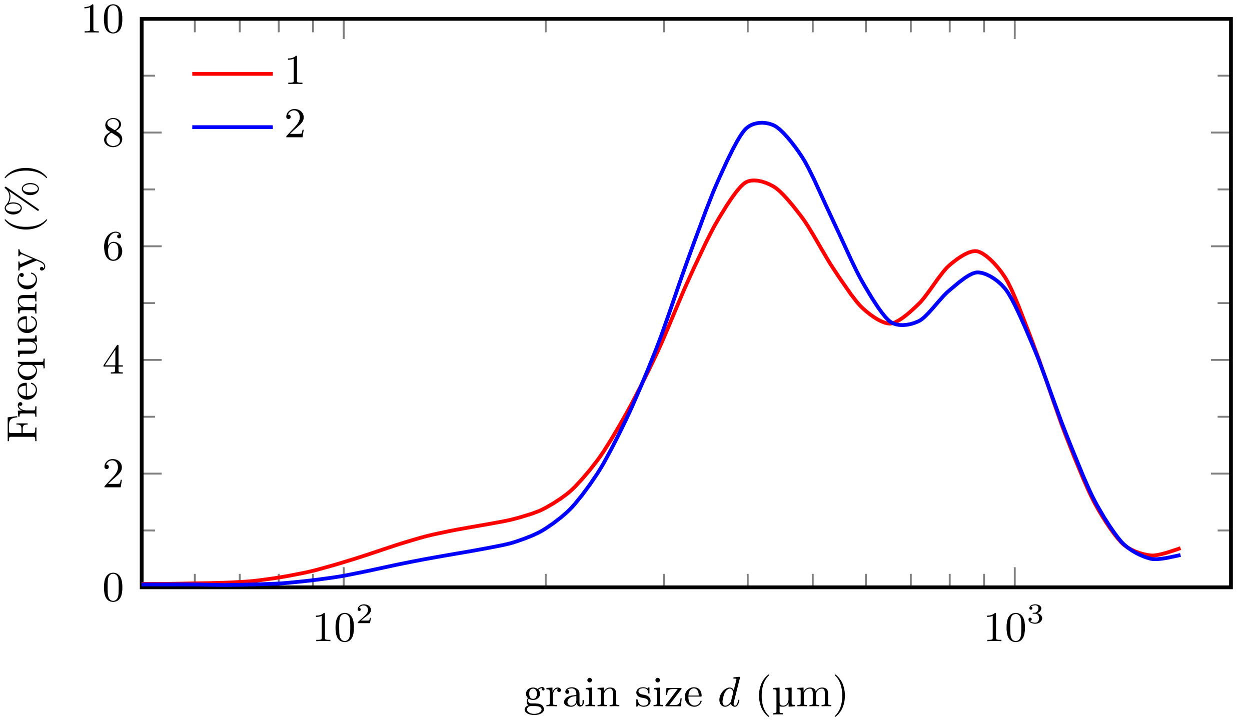

FIG. S7 Our measurements of the GSDs from Sossusvlei, Namibia (see Fig. 1).

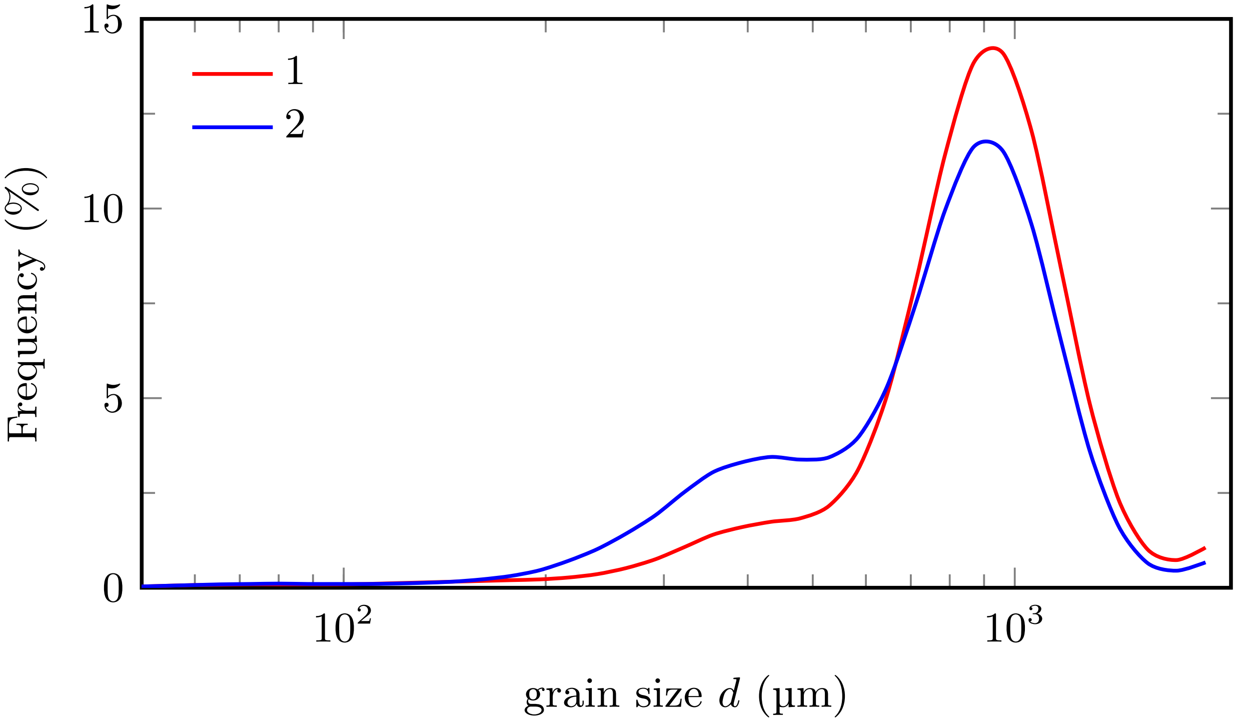

FIG. S8 Our measurements of the GSDs from Ladakh, India (see Fig. S9f).

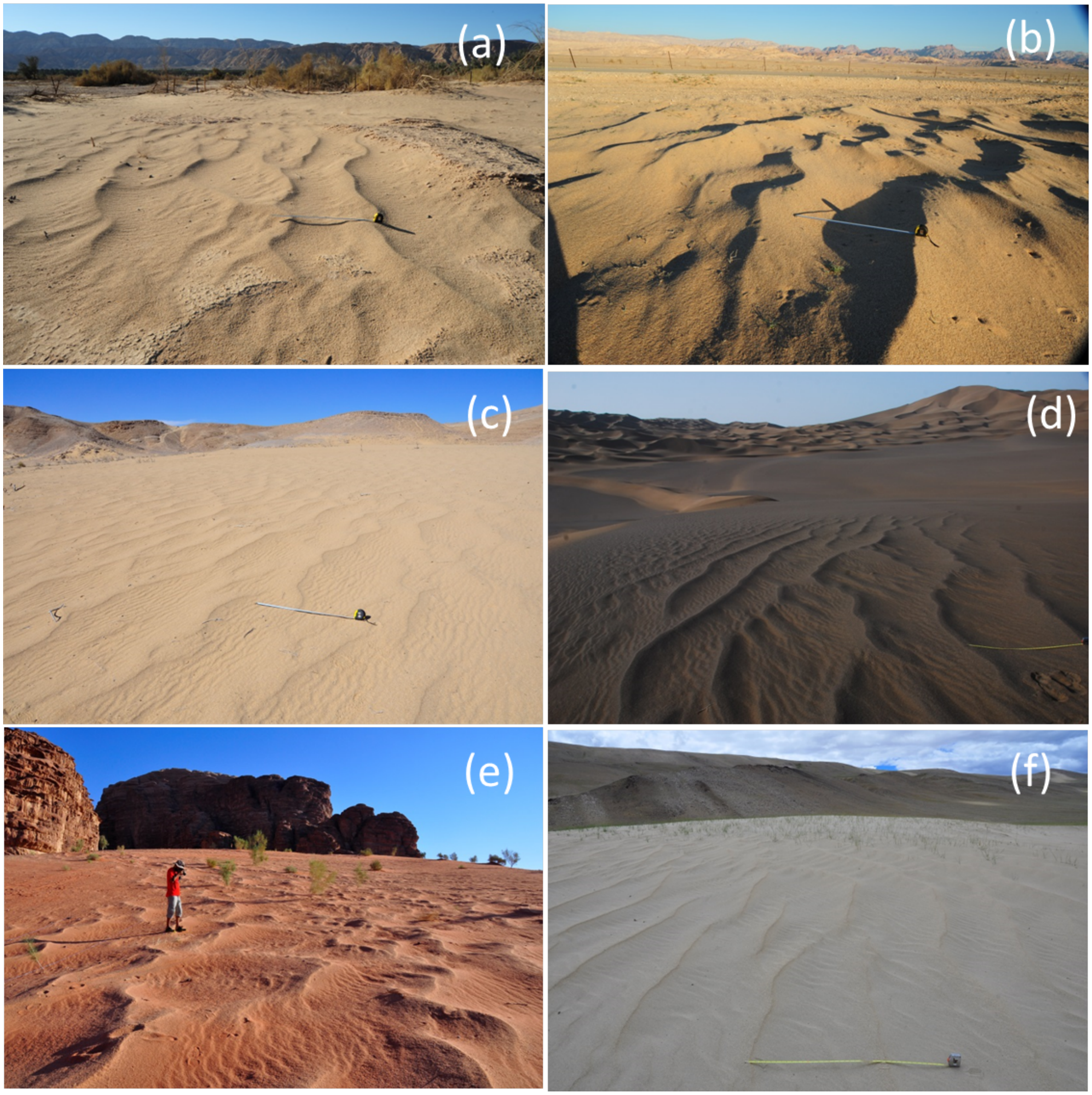

Fig. S9 **Examples of megaripples whose surface GSDs were analyzed.** (a) Ktora in the Arava Valley, Israel. (b) Kasuy in the southern Negev Desert, Israel. (c) Yahel in the Arava Valley. (d) Shanshan Desert, also known as Kumtagh Desert in Xinjiang, China. (e) Wadi Rum, Jordan. (f) North of Tso Moriri lake in Ladakh, India, at altitude 4522 m above sea level. The scales indicate 1 m length. Superimposed smaller impact ripples can be seen in the megaripple troughs.

REFERENCES

[a] Corresponding authors: Klaus Kroy (klaus.kroy@uni-leipzig.de), Thomas Pähtz (0012136@zju.edu.cn), Itzhak Katra (katra@bgu.ac.il)

[1] T. Pähtz, Y. Liu, Y. Xia, P. Hu, Z. He, and K. Tholen, "Unified model of sediment transport threshold and rate across weak and intense subaqueous bedload, windblown sand, and windblown snow," J. Geophys. Res. Earth Surf. **126**, e2020JF005859 (2021).

[2] M. Lämmel, K. Dzikowski, K. Kroy, L. Oger, and A. Valance, "Grain-scale modeling and splash parametrization for aeolian sand transport," Phys. Rev. E **95**, 022902 (2017).

[3] D. Beladjine, M. Ammi, L. Oger, and A. Valance, "Collision process between an incident bead and a three-dimensional granular packing," Phys. Rev. E **75**, 061305 (2007).

[4] H. Yizhaq, G. Bel, S. Silvestro, T. Elperin, J. Kok, M. Cardinale, A. Provenzale, and I. Katra, "The origin of the transverse instability of aeolian megaripples," Earth. Planet. Sci. Lett. **512**, 59–70 (2019).

[5] I. Katra and H. Yizhaq, "Intensity and degree of segregation in bimodal and multimodal grain size distributions," Aeolian Res. **27**, 23–34 (2017).

[6] H. Yizhaq, I. Katra, O. Isenberg, and H. Tsoar, "Evolution of megaripples from a flat bed," Aeolian Res. **6**, 1–12 (2012).

[7] G. Qian, Z. Dong, Z. Zhang, W. Luo, and J. Lu, "Granule ripples in the kumtagh desert, china: Morphology, grain size and influencing factors," Sedimentology **59**, 1888–1901 (2012).

[8] J. A. Gillies, W. G. Nickling, M. Tilson, and E. Furtak-Cole, "Wind-formed gravel bed forms, wright valley, antarctica," J. Geophys. Res. Earth Surf. **117** (2012).

[9] D. J. Jerolmack, D. Mohrig, J. P. Grotzinger, D. A. Fike, and W. A. Watters, "Spatial grain size sorting in eolian ripples and estimation of wind conditions on planetary surfaces: Application to meridiani planum, mars," J. Geophys. Res. **111** (2006).

[10] T. Gough, C. Hugenholtz, and T. Barchyn, "Eolian megaripple stripes," Geology **48**, 1067–1071 (2020).

[11] R. Bagnold, The physics of blown sand and desert dunes (Methuen, London, 1941).

[12] M. J. Selby, R. B. Rains, and R. W. P. Palmer, "Eolian deposits of the ice-free victoria valley, southern victoria land, antarctica," New Zeal. J. Geol. Geophys. **17**, 543–562 (1974).

[13] N. P. Mountney and A. J. Russell, "Sedimentology of cold-climate aeolian sandsheet deposits in the askja region of northeast iceland," Sediment. Geol. **166**, 223–244 (2004).

[14]C. Hong, L. Huiru, F. Yi, Z. Xueyong, L. Jifeng, K. Liqiang, L. Bo, and L. Chenchen, "Particle size characteristics of aeolian ripple crests and troughs," Sedimentology **65**, 1859–1874 (2018).

[15]C. McKenna Neuman and O. Bédard, "A wind tunnel investigation of particle segregation, ripple formation and armouring within sand beds of systematically varied texture," Earth Surf. Process. Landf. **42**, 749–762 (2017).
